# Assessment of volatile component stability in Guanxin Jieyu granules through gas chromatography in accordance with Q14 guidelines

**DOI:** 10.1038/s41598-025-31589-8

**Published:** 2025-12-25

**Authors:** Liquan Chen, Ting Zhang, Ningji Fang, Haofang Wan, Yu He, Haitong Wan

**Affiliations:** 1 College of Basic Medical Sciences, School of Basic Medicine Sciences, Zhejiang Chinese Medical University, 548 Binwen Road, Hangzhou, 310053 Zhejiang People’s Republic of China; 2 Zhejiang Key Laboratory of Chinese Medicine for Cardiovascular and Cerebrovascular Disease, Hangzhou, 310053 Zhejiang People’s Republic of China; 3 School of Pharmaceutical Sciences, Zhejiang Chinese Medical University, Hangzhou, 310053 Zhejiang People’s Republic of China; 4 Academy of Chinese Medical Sciences, Zhejiang Chinese Medical University, Hangzhou, 310053 Zhejiang People’s Republic of China; 5 Academy of Chinese Medical Sciences, Henan University of Chinese Medicine, Zhengzhou, 450046 Henan People’s Republic of China

**Keywords:** Experimental design, Borneol, Gas chromatography, Development of analytical methods, Accelerated destructive degradation tests, Drug discovery, Medical research

## Abstract

Guanxin Jieyu Granules, a novel traditional Chinese medicine (TCM) in development, targets stable angina pectoris with accompanying anxiety. To improve stability, its volatile oil components—including borneol—are stabilized via β-cyclodextrin inclusion. In compliance with ICH Q14 guidelines, a gas chromatography (GC) analytical method was developed, incorporating knowledge management, risk management, experimental design, and results analysis, as well as performance evaluation of the analytical method. Accelerated Destructive Degradation Tests (ADDTs) using this method revealed a dramatic stability enhancement from β-cyclodextrin encapsulation. At 25 °C over 730 days, the inclusion complex showed only a 1.10% probability of failure (defined as ≥ 5% content loss), compared to 88.50% for the physical mixture. These results underscore the critical role of cyclodextrin inclusion in preserving volatile TCM constituents.

## Introduction

Borneol (1,7,7-Trimethylbicyclo[2.2.1]heptan-2-ol, C10H18O) has a molecular weight of 154.25 and a boiling point of 212°C^1^. The literature has linked borneol to potential relief from pain and itch. A randomized controlled clinical statistical analysis indicated that a sublingual spray formulation combining essential oils with Borneol can effectively alleviate chest pain symptoms^2^. Furthermore, one clinical study demonstrated that a traditional Chinese medicine formula containing Borneol—Suxiaojiuxin pill—can treat chronic stable angina pectoris^3^. Borneol has also been identified in research as having anticoagulant effects, which may possess anti-thrombotic properties^4^. In a mouse pain model study^5^, TRPM8 was suggested as a potential target for the pain-relieving effects of Borneol. In another mouse itch model^6^, TRPM8 was also identified as a potential target for Borneol in providing itch relief.

Forsythia fruit (Forsythia suspensa (Thunb.) Vahl) and Dalbergia (Dalbergia odorifera T. Chen) have been traditionally utilized in Chinese medicine to address myocarditis. Cardiac hypertrophy was induced in C57BL/6 mice through subcutaneous injection of norepinephrine (NE). Administration of Forsythia at a dosage of 100 mg/kg via intraperitoneal injection for 15 days significantly improved cardiac function, alleviated histopathological changes, and reduced cardiac hypertrophy along with markers of hypertrophy (ANP, BNP, and β-MHC)^7^. Additionally, in an in vitro model using rat cardiomyoblasts (H9c2) stimulated with NE, Forsythia effectively reduced the infiltration of CD68-positive macrophages and the expression of pro-inflammatory genes (IL-1β, IL-6, and TNF-α) in the left ventricular tissue. This was associated with a mitigation of NE-induced cardiac hypertrophy and inflammation through the inhibition of the p38 MAPK/ERK1/2 and AKT/NF-κB signaling pathways^7^. In another study utilizing an animal model of ApoE-/- mice fed a high-fat diet (HFD), Dalbergia demonstrated the ability to regulate lipid levels and reduce intimal hyperplasia in atherosclerotic lesion models^8^. The volatile oils of Dalbergia exhibit significant cardioprotective effects against myocardial ischemia, with trans-nerolol and its oxidized derivatives identified as the primary pharmacodynamic components. Trans-nerolol confers protection against myocardial ischemia (MI) through mechanisms involving the inhibition of the Cytc and caspase signaling pathways^9^. Furthermore, research on a traditional Chinese medicine formula containing sandalwood volatile oil, using a mouse model with left anterior descending coronary artery ligation to induce myocardial ischemia, indicated that this formulation promotes mitochondrial biogenesis (involving PGC-1α, Nrf1, and TFAM) and mitochondrial fusion (MFN-2 and OPA1), while inhibiting mitochondrial fission (as evidenced by the phosphorylation of Drp1 at Ser616) in vivo. It indicates that the cardioprotective effects may be associated with the enhancement of mitochondrial biogenesis and dynamic homeostasis^10^. Moreover, essential oils with aromatic properties have been shown to influence mood. Studies indicate that plant essential oils facilitate neurogenesis, modulate hormone levels, activate various brain regions, and induce biochemical changes in the blood, ultimately affecting emotions and mood. They can inhibit sympathetic nervous system activity and exert their effects through the olfactory and respiratory systems, suggesting potential for mood regulation and anxiety treatment^11,12^. We are developing a new class of drugs that incorporates borneol and essential oils as raw materials for the treatment of stable angina pectoris with accompanying anxiety.

An evaluated, correct analytical approach is necessary^13^. Analytical Quality by Design (AQbD) extends the benefits of the Quality by Design (QbD) approach to the development of analytical methods. In this regard, the ICH Q14 guideline provides a structured framework for the development and validation of these methods. The guideline aims to establish control strategies to ensure that analytical methods consistently remain within their validated parameters throughout their lifecycle, while also supporting the continuous optimization of these methods^14^. Based on the product attributes, we have evaluated the analytical methods and decided to use gas chromatography for the quantitative analysis of volatile substances. Conducting Risk identification and assessment(Table 1), as well as managing the Analytical target profile for gas chromatography methods(Table 2).

**Table 1:** Risk identification and assessment.

Source of risk	System adaptability-related impact	Risk level	References
Chromatographic column	Resolution, retention time, peak area, theoretical plates	High	^15–17^
Injection volume	Peak symmetry, peak area	Medium	^16^
Needle wash program	Peak symmetry	Low	
Split ratio	Retention time, peak symmetry, peak area, theoretical plates	High	^15^
Carrier gas type	Resolution, retention time	Low	^16,18,19^
Column flow rate	Resolution, retention time, peak area, theoretical plates	High	^15, 19^
Injection port temperature	Retention time, resolution	Medium	
Column temperature	Peak symmetry, resolution, retention time, theoretical plates	High	^15,17,19^
Detector type	Retention time, peak area	Medium	^16, 20^
Detector temperature	Retention time, peak area	Medium	
Standard/sample concentration	Peak symmetry, peak area	High	^15, 20^
Diluent type	Resolution, retention time	Medium	^15, 20^
Sample filtration	Peak area	Low	

**Table 2:** Analytical target profile.

Performance characteristics	Performance characteristics	Acceptance criteria	Is it a CQA?	References
Resolution	Resolution between the main peak and impurity peak ≥ 2.0	General requirements of the Ch.P 2020	Yes	^21^
Relative retention time	As short as possible	Improve detection efficiency	No	
Tail factor	Between 0.95 and 1.05	General requirements of the Ch.P 2020	No	^21^
S/N	QL > 10	General requirements of the Ch.P 2020	No	^21^
Peak Area	None	None	No	
Theoretical plate count	≥ 2000	General requirements of the Ch.P 2020	No	^21^

CQA: critical quality attribute.

QL: Quantitation limit.

Ch.P 2020: Pharmacopoeia of People’s Republic of China 2020.

## Materials and methods

### Sample collection

The herbal materials were purchased from Hangzhou Huadong Traditional Chinese Medicine Pieces Co., Ltd: Forsythia fruit (20241130), Dalbergia (20241016), and Borneol (20240507). The synthesis of inclusion compounds at the pilot scale is entrusted to Hangzhou Huiyuan Industrial Co., Ltd. All medicinal materials have been tested by both parties and comply with the requirements of the 2020 edition of the Chinese Pharmacopoeia. The procurement source of beta-cyclodextrin has a production license for food and pharmaceuticals.

### Reagents

Methanol (≥ 99.9%) and ethanol (≥ 99.9%) were purchased from Hangzhou Marychem Co., Ltd. Ethyl acetate and 0.22 μm organic phase filter heads are sourced from Hangzhou Hehui Chemical Co., Ltd. Beta-cyclodextrin is purchased from Anhui Shanhe Pharmaceutical Excipients Co., Ltd.

### Equipment

The analytical balance is from METTLER TOLEDO, model XS205 (0.01 –120 g); the non-precision balance is from ISMART, model IS1000-2 C (0.01 –1100 g).

Multi-head Magnetic Stirring Heater from Changzhou Guohua Electric Co., Ltd. Model HJ-6 A.

Medical Refrigerated Box from Haier Biomedical Model HYC-1031GD.

Electric Blast Drying Oven from BOXUN Model GZX-9140MBE.

Volumetric flask, pipette and micropipette (Dragon-Lab, 100–1000 µL, 10–100 µL) were calibrated by Hangzhou Shenzhou Instrument Calibration Company.

The gas chromatography-flame ionization detection (GC-FID) system (HS-20, Shimadzu) was equipped with an automatic sampler (AOC20i) and an FID detector (GC2010). Separation was achieved using an Agilent DB-FFAP capillary column (30 m length, 0.25 mm internal diameter, 0.20 μm film thickness).

Mass spectrometric analysis was performed using a GCMS-QP2010 Plus mass spectrometer.

Field emission scanning electron microscopy (FE-SEM) images were obtained using a Hitachi SU8010 instrument.

### Preparation for standard solutions

Borneol standard (MACKLIN, concentration 98%, lot # C16995655, B803368-200 mg): An accurate weight of 10 mg of borneol (to the nearest 0.01 mg) was dissolved in 5 mL of ethyl acetate. After complete dissolution, the solution was transferred to a 10 mL volumetric flask and diluted to 10 mL in the volumetric flask, resulting in a standard solution with a concentration of 1 mg/mL (1,000 µg/mL). The solution was mixed thoroughly and filtered through a 0.22 μm organic phase filter membrane. The filtrate was then transferred into capped sample vials to prevent solvent evaporation, making the sample ready for analysis.

### Preparation for sample

The essential oil was extracted by refluxing Forsythia and Dalbergia in a 4:3 ratio for 7 h, collecting it from the volatile oil extractor. The essential oil and Borneol were dissolved in 95% ethanol, and a saturated cyclodextrin solution was prepared separately. The two solutions were then mixed in a 1:5 ratio and stirred at room temperature at 250 rpm for 1 h. After refrigeration for 24 h, the mixture was dried in an environment at 60 degrees Celsius, then crushed and sieved through an 80 mesh screen for collection.

An accurate weight of the sample (to the nearest 1 mg) is placed in a round-bottom flask. Ten times the sample volume of water is added, and the essential oil extractor is connected. The mixture is heated and refluxed for 1 h, then allowed to cool. The liquid from the essential oil extractor is collected and mixed with 5 mL of ethyl acetate for liquid-liquid extraction. The upper liquid phase is collected and then transferred to a 10 mL volumetric flask, where it is brought to volume with ethyl acetate. The solution is mixed thoroughly and filtered through a 0.22 μm organic phase filter membrane. The filtrate was then transferred into capped sample vials to prevent solvent evaporation, making the sample ready for analysis^22^.

### Inclusion process

The inclusion complexes were produced using a mixer based on electromagnetic actuation(Shanghai Meiyingpu Instrument Manufacturing Co., Ltd. 84 − 1).

Pilot-scale samples (batch 250501) for accelerated degradation studies were provided by Zhejiang Huisong Pharmaceuticals Co., Ltd.

### Headspace GC‑FID analysis

The carrier gases used were hydrogen, nitrogen, and air. The injector temperature was maintained at 220 °C, while the detector temperature was set at 240 °C. The injection volume was 1 µL, and an appropriate needle wash procedure was performed before and after the injection.

### GC-MS analysis

Samples were analyzed using a Shimadzu AOC-20i autosampler coupled with a GC-2010 gas chromatograph and GCMS-QP2010 Plus mass spectrometer. The syringe was rinsed twice with presolvent, four times with solvent, and twice with the sample. Injection was performed in normal split mode with a split ratio of 20, and the injection port temperature was set at 220 °C. Plunger speeds were set high for suction and medium for injection, with an injection dwell time of 0.3 s. Helium was used as the carrier gas under linear velocity control at 61.3 kPa, maintaining a column flow of 1.00 mL/min and a purge flow of 3.0 mL/min.

The oven temperature was programmed starting at 70 °C, ramped to 140 °C over 15 min and held for 5 min, then ramped to 200 °C over 20 min and held for 3 min. The mass spectrometer ion source and interface temperatures were maintained at 220 °C and 240 °C, respectively, with a solvent cut time of 2 min. Data were acquired in scan mode from m/z 40 to 550 with an event time of 0.5 s. Samples were equilibrated for 3 min before injection^22^.

### Field emission scanning electron microscopy (FE-SEM) imaging

Use double-sided carbon tape to mount a small amount of the powder onto a conductive stub. A thin layer of gold is then uniformly sprayed to enhance its conductivity. The mounted sample is then placed into the FE-SEM chamber for imaging.

### Two-level factorial design development analysis method

Based on the preliminary test results, experimental operability, and the degree of factor risk, four factors were selected: A column temperature, B column flow rate, C split ratio, and D sample dilution factor. The response variables for the analysis of the Borneol peak include retention time, front peak separation, back peak separation, symmetry factor, theoretical plate number, and peak area. A 2^4^ factorial design was employed, with only the coded levels (-1 to + 1) provided, as the actual levels needed to remain proprietary. Table 3 presents the design matrix with the selected factors at low (-1) and high (+ 1) levels for 16 experimental runs. Additionally, it is essential to determine the analytical quality control strategy, which includes the Analytical Design Space and the Method Operable Design Region^13^.

**Table 3:** Two-level factorial design.

Std	Run	Column temperature	Gas flow rate	Flow ratio	Multiple of dilution
		℃	mL/min	times	times
12	1	1	1	-1	1
14	2	1	-1	1	1
10	3	1	-1	-1	1
6	4	1	-1	1	-1
1	5	-1	-1	-1	-1
11	6	-1	1	-1	1
7	7	-1	1	1	-1
2	8	1	-1	-1	-1
13	9	-1	-1	1	1
5	10	-1	-1	1	-1
4	11	1	1	-1	-1
3	12	-1	1	-1	-1
16	13	1	1	1	1
9	14	-1	-1	-1	1
15	15	-1	1	1	1
8	16	1	1	1	-1

Table 4 the design power is used to assess the probability that a factor variation in the model results in a positive outcome, power that exceeds 80% probability of seeing the desired difference.

**Table 4:** The design power.

Name	Units	Delta (signal)	Sigma (noise)	Signal/noise	Power for A	Power for B	Power for C	Power for D
Retention time	min	2	1	2	95.3%	95.3%	95.3%	95.3%
Front resolution		2	1	2	95.3%	95.3%	95.3%	95.3%
Back resolution		2	1	2	95.3%	95.3%	95.3%	95.3%
Symmetry factor		2	1	2	95.3%	95.3%	95.3%	95.3%
Theoretical plates		2	1	2	95.3%	95.3%	95.3%	95.3%
Peak area		2	1	2	95.3%	95.3%	95.3%	95.3%

### Evaluation of analytical procedure performance characteristic

The chromatographic method was evaluated in terms of key performance parameters, including precision, accuracy, and linearity. The precision assessment focused on intra-day repeatability, using standard solutions of known concentrations prepared at five different concentration levels, each analyzed in triplicate to establish the calibration curve. The percentage recovery was also evaluated to assess the method’s accuracy.

#### Precision (repeatability)

Six consecutive replicate injections were performed, and the relative standard deviation (RSD) based on the FID peak area was calculated using the following Eq. 2^1^:

1 $$\:\begin{array}{c}\mathrm{R}\mathrm{S}\mathrm{D}\hspace{0.17em}\left(\%\right)=\left(\frac{s}{\stackrel{-}{x}}\right)\times\:100\end{array}$$

2 $$\:\begin{array}{c}\stackrel{-}{x}=\frac{1}{n}{\sum\:}_{i=1}^{n}{x}_{i}=\frac{{x}_{1}+{x}_{2}+\cdots\:+{x}_{n}}{n}\end{array}$$

3 $$\:\begin{array}{c}s=\sqrt{\frac{1}{n-1}{\sum\:}_{i=1}^{n}{\left({x}_{i}-\stackrel{-}{x}\right)}^{2}}\end{array}$$

#### Linearity

Five different concentrations of standard solutions were used for linear regression. For each concentration, three parallel experiments were performed. Quantification of the analyte in the samples was based on the FID peak area, with the average peak area of the three replicates calculated. The mean peak areas of the five concentrations were plotted on the y-axis, while the known added mass of the analyte was plotted on the x-axis. A least squares linear regression was conducted to fit the data, and the model fit was evaluated using metrics including the coefficient of determination (*R²*) and the *p-values* for both the intercept and the slope. The linear range was thus characterized. Additionally, the method’s sensitivity was evaluated by calculating the detection limits (DL) and quantification limits (QL) using the residual standard deviation $\:\sigma\:$ of the regression and the slope $\:\mathrm{S}$ of the calibration curve, according to the formulas^21^:

4 $$\:\begin{array}{c}LOD=\frac{3.3\sigma\:}{S}\:\end{array}$$

5 $$\:\begin{array}{c}LOQ=\frac{10\sigma\:}{S}\end{array}$$

#### Accuracy

Accuracy was assessed through spike recovery. The added spike concentration was determined by preparing standards for the analyte through precise weighing (to the nearest 0.1 mg). For preliminary analysis, the first three headspace samples were evaluated without the addition of standards, and the measured concentration in the unspiked samples was calculated using the average value. For the measured concentration in the spiked samples, standards were mixed with the samples at a 1:1 ratio relative to the target analyte, and headspace vials were prepared as described in “Preparation for sample”. The RSD value was calculated based on the recoveries from six sets of data. The Recovery (%) was computed using the formula^21^:

6 $$\:\begin{array}{c}Recovery\hspace{0.17em}\left(\%\right)=\left(\frac{{C}_{\mathrm{s}\mathrm{p}\mathrm{i}\mathrm{k}\mathrm{e}\mathrm{d}}-{C}_{\mathrm{u}\mathrm{n}\mathrm{s}\mathrm{p}\mathrm{i}\mathrm{k}\mathrm{e}\mathrm{d}}}{{C}_{\mathrm{a}\mathrm{d}\mathrm{d}\mathrm{e}\mathrm{d}\text{}}}\right)\times\:100\end{array}$$

The RSD value was calculated using formulas pertaining to “Precision (repeatability)”.

### Accelerated destructive degradation tests

The stability study was conducted in accordance with ICH Q2(R1) guidelines^23^, employing accelerated destructive degradation testing. Samples were packaged in three-layer PET/AL/PE composite film bags (2 g fill weight per bag) and heat-sealed at 200 °C. Gas chromatography (GC) analysis was performed at 2-day intervals on precisely weighed samples to quantify Borneol content. A physical mixture of Borneol and β-cyclodextrin was processed identically as a reference material. Three replicate measurements were taken at each time point for two temperature conditions. The detailed Test Plan can be found in Table 5.

**Table 5:** Test plan.

Temp (°C)	Days aged					
	0	2	4	6	8	10
60	3	3	3	3	3	3
80	3	3	3	3	3	3

The observed response $\:{y}_{ijk}$​ is modeled as:

7 $$\:\begin{array}{c}{y}_{ijk}=D\left({\tau\:}_{i},{\chi\:}_{j},\beta\:\right)+{\epsilon\:}_{ijk}\end{array}$$

where $\:\mathcal{D}\left({\tau\:}_{i},{\chi\:}_{j},\beta\:\right)\:$represents the underlying diffusion process. $\:\beta\:$ denotes the fixed model parameters, and $\:{\epsilon\:}_{ijk}\:$corresponds to the residual term. Time and Temperature are transformed using standard diffusion kinetics:

8 $$\:\begin{array}{c}{\tau\:}_{i}=\sqrt{{\mathrm{Days}}_{i}}\end{array}$$

9 $$\:\begin{array}{c}{\chi\:}_{j}=\frac{{\mathrm{E}}_{\alpha\:}}{\mathrm{R}}\cdot \:\frac{1}{{{\deg {\rm C}}}_{j}+273.15}=\frac{{\mathrm{E}}_{\alpha\:}}{\mathrm{R}}\cdot \:\frac{1}{{\mathrm{T}}_{j}}\end{array}$$

Here, $\:i$ indexes time groups, $\:j$ indexes temperature groups, and $\:k$ indexes measurement replicates. $\:{\mathrm{E}}_{\alpha\:}$ represents the activation energy, $\:\mathrm{R}$ is the gas constant, and $\:{\mathrm{T}}_{j}$ denotes the explanatory variable temperature (converted to Kelvin). Residual $\:{\epsilon\:}_{ijk}$ are assumed to follow a normal distribution: $\:{\epsilon\:}_{ijk}\sim\:\mathcal{N}\left(0,{\sigma\:}^{2}\right)$, Standardized Residuals are defined as: $\:\mathcal{Z}=\frac{{\epsilon\:}_{ijk}}{\sigma\:}\sim\:\mathcal{N}\left(\mathrm{0,1}\right)$ ^24,25^.

### Software and code

Data collection was performed using the Shimadzu GC Solution system, with all raw data complying with auditing regulatory standards. Consistent integration parameters were applied throughout the entire experiment. Randomizing run order, data analysis, isosurface generation, and MODR planning and validation for the two-level factorial design was carried out using Stat-Ease^®^ 360 software (version 23.1, Stat-Ease, Inc., Minneapolis, MN, USA). Accelerated destructive degradation test data were analyzed using JMP^®^ (version 17, SAS Institute Inc., Cary, NC, USA).

## Results and discussion

### Characterization of inclusion complexes: volatile components, crystallinity, and microstructure analysis

**Fig. 1:**
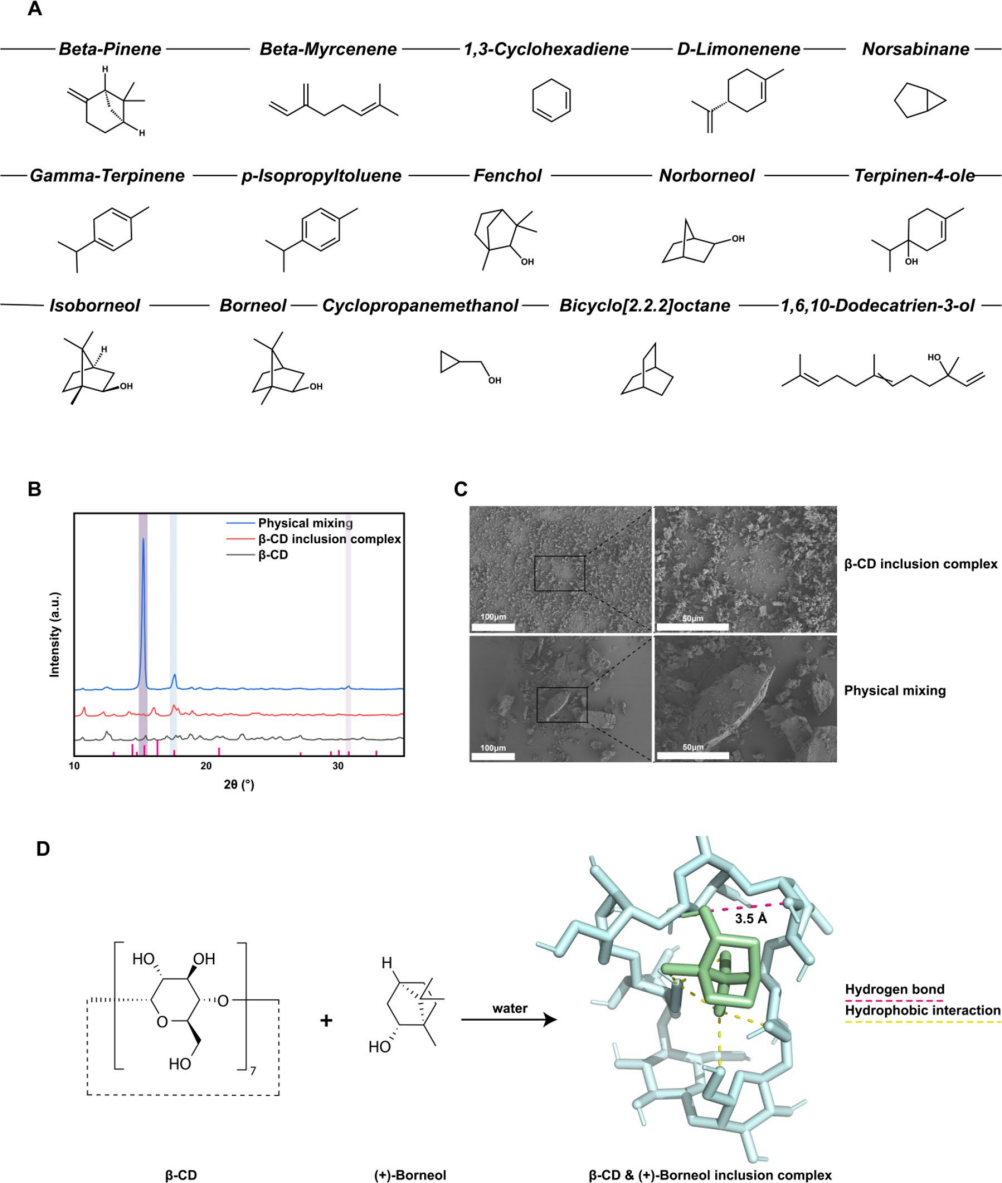
Characterization of inclusion complexes: volatile components, crystallinity, and microstructure analysis. (**A**) GC-MS analysis of volatile components in Guanxin Jiuyu Granules, identifying at least 15 compounds. Characterization includes: (**B**) X-ray diffraction (XRD) for crystallinity, (**C**) field emission scanning electron microscopy (FESEM) for microstructure, (**D**) β-Cyclodextrin forms a complex with (+)-borneol through hydrogen bonding and hydrophobic interactions.

As shown in Fig. 1, the inclusion complexes contain at least 15 volatile components. The characterization of the inclusion complex was conducted using X-ray diffraction (XRD) to assess its crystallinity and field emission scanning electron microscopy (FESEM) to examine its microstructure.

Figure 1A shows the GC–MS results were presented to analyze the volatile components of Guanxin Jieyu Granules. At least 15 chromatographic peaks with peak areas ≥ 0.42% were detected. For each peak, the top five most likely compounds were retrieved from the NIST17s database, and the corresponding structures of the most probable compounds were downloaded from PubChem.

Figure 1B shows the X-ray diffraction (XRD) patterns of the physical mixing, the β-CD inclusion complexes, and the β-CD alone. The diffraction angles were scanned from 2θ = 10° to 35°. The red markers on the x-axis correspond to a standard card for(+)borneol. Shaded regions highlight three peaks in the physical mixing that align with the positions of the standard card, whereas the β-CD inclusion complexes do not exhibit these peaks, and there is no interference from β-CD. This suggests a substantial alteration in the crystalline structure.

Figure 1C presents FESEM images of β-CD inclusion complexes and physical mixtures acquired at two different magnifications to compare their morphology and particle size. At 300× magnification (100 μm scale) and 1000× magnification (50 μm scale), the β-CD inclusion complexes appear as relatively dispersed particles. Following β-CD coating, the inclusion complexes demonstrate improved dispersion, with a finer and more uniform morphology. In contrast, the physical mixture sample exhibits an aggregated, crystalline structure with distinct particle size variations, indicating significant differences in particle size distribution between the two types of samples.

β-Cyclodextrin is formed through the cyclization of seven glucose units, resulting in a cyclic oligosaccharide. The upper end (the larger opening) is characterized by secondary hydroxyl groups at C2 and C3, while the lower end (the smaller opening) comprises the primary hydroxyl group at C6. Due to the shielding effect of the C–H bonds, a hydrophobic region is created within the cavity^26,27^. Figure 1D shows the lowest-energy conformer from computer simulations for the binding of β-cyclodextrin with (+) borneol, with an O···O distance of 3.5 Å denoted by a red dashed line in the figure. This distance exceeds the typical range for O–H···O hydrogen bonds, which generally falls between 1.40 Å and 2.00 Å^28^, indicating that hydrogen bonding is relatively weak. The yellow dashed lines represent the distances between the hydrophobic group of cyclic oligosaccharide and the hydrophobic group of (+) borneol, highlighting the role of hydrophobic interactions in their association^29^.

### Two-level factorial design

**Fig. 2:**
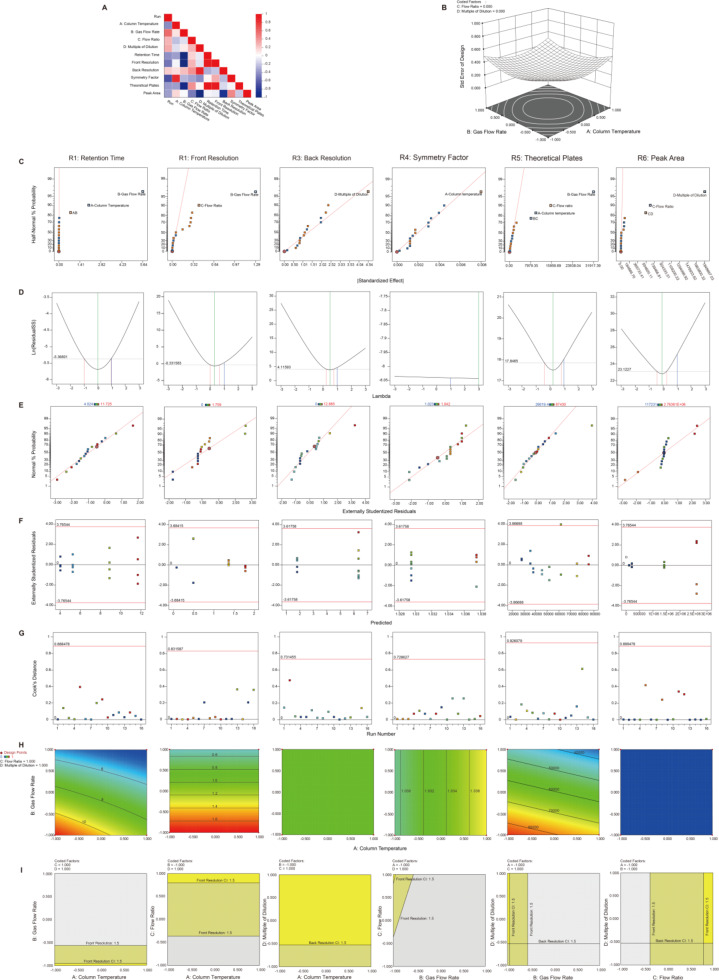
The diagnostic plots, analytical design space, and method operable design region of two-level factorial deign. (**A**) Correlation heatmap and response of the factors in Model. (**B**) Standard error of the model estimates. A series of diagnostic plots (C-G) designed to evaluate the statistical validity of the model: (**C**) The Half-Normal Plot illustrates factor effects, with orange indicating positive influences and blue indicating negative influences. (**D**) The Box-Cox Plot for Power Transforms, based on the optimal lambda, used to determine the appropriate power law transformation. (**E**) The Normal Plot of Residuals to assess whether the residuals follow a normal distribution and to evaluate if data transformation is necessary. (**F**) The Residuals vs. Predicted plot examining the assumption of constant variance, indicating whether residuals exhibit random scatter. (**G**) Cook’s Distance measuring the influence of individual observations on the regression model. The Analytical Design Space and Method Operable Design Region: (**H**) The Analytical Design Space. (**I**) Method Operable Design Region, defining the feasible operational conditions for gas chromatography. The constraints include Front Resolution and Back Resolution both greater than 1.5, and Theoretical Plate number exceeding 2000. The figure shows the non-operational space in gray, the one-sided 95% confidence interval in buff, and the feasible operational space in bright yellow, with specific spatial constraints annotated.

Using Stat-Ease^®^ 360 software (version 23.1) for a 24-factor factorial design, the regression models and ANOVA were fitted and analyzed. The experimental design comprised 16 runs with varying levels of A-Column Temperature, B-Gas Flow Rate, C-Flow Ratio, and D-Multiple of Dilution, aiming to identify optimal gas chromatography parameters. The responses included the analysis of the Borneol peak, encompassing Retention time, Front peak resolution, Back peak resolution, Symmetry factor, Theoretical plate number, and Peak area. Based on the Half-normal probability plot in Fig. 2C, all significant effects were selected. The unacceptable factor levels were then used to estimate the remaining errors.

**Table 6:** Analysis of variance (ANOVA).

Response	Source	*P* value	Significance	Std. dev.	*R*²
Retention time	Model	< 0.0001	Significant	0.0199	1.0000
	A-column temperature	< 0.0001			
	B-gas flow rate	< 0.0001			
	AB	< 0.0001			
Front resolution	Model	< 0.0001	Significant	0.3341	0.8346
	B-gas flow rate	< 0.0001			
	C-flow ratio	0.0278			
Back resolution	Model	0.0066	Significant	2.85	0.4447
	D-multiple of dilution	0.0066			
Symmetry factor	Model	0.0049	significant	0.0048	0.4429
	A-column temperature	0.0049			
Theoretical plates	Model	< 0.0001	Significant	2481.02	0.9883
	A-column temperature	< 0.0001			
	B-gas flow rate	< 0.0001			
	C-flow ratio	< 0.0001			
	BC	< 0.0001			
Peak area	Model	< 0.0001	Significant	83779.96	0.9949
	C-flow ratio	< 0.0001			
	D-multiple of dilution	< 0.0001			
	CD	< 0.0001			

The ANOVA results, presented in Table 6, showed that all models were highly significant, with *p*-values less than 0.01. Specifically, A-Column Temperature and B-Gas Flow Rate, as well as their interaction (AB), significantly affected retention time. B-Gas Flow Rate and C-Flow Ratio influenced front peak resolution. D-Multiple of Dilution impacted back peak resolution. A-Column Temperature significantly affected the symmetry factor. The interaction between B-Gas Flow Rate and C-Flow Ratio (BC) influenced the theoretical plate number, while C-Flow Ratio and D-Multiple of Dilution (CD) affected peak area.

Furthermore, aside from the effects of back peak resolution and symmetry factor, the determination coefficient (*R²*), along with the Std. Dev., indicated that the regression models provided a good fit to the data, with low associated error, accurately reflecting the actual variations observed in the experimental groups. Coded Equation in Table 7.

**Table 7:** Coded equation.

Coded equation
Retention time = 7.47–1.01*A-2.82*B + 0.3934*AB
Front peak resolution = 0.9311 − 0.6442*B + 0.2069*C
Back peak resolution = 4.08 + 2.27*D
Symmetry factor = 1.03 + 0.0040*A
Theoretical plate number = 52714.10-5001.77*A-15958.69*B + 7828.40*C-4078.89*BC
Peak area = 1.09E + 06-3.249E + 05*C-9.243E + 05*D + 2.747E + 05*CD

As illustrated in Fig. 2, the model evaluation results are presented, including (A) the model’s correlation heatmap used to illustrate the relationships between every factor and every response. Adjacent color bars indicate the direction and strength of correlation, where red denotes positive correlations and blue denotes negative correlations; darker colors correspond to stronger correlations. (B) The standard error of the model. The standard error quantifies the amount of error transmitted to the response from variation in factor settings. Propagation of Error (POE) is assessed using a second-order hierarchical response surface model and the estimated standard deviations for the numeric factors. Essentially, the POE approach involves applying partial derivatives to locate flat regions on the response surface. In the plots, the standard error at the center is markedly lower than in the surrounding regions.

A series of diagnostic plots (C–G) are designed to evaluate the statistical validity of the model: (C) The Half-Normal Plot, the primary model-selection tool for factorial designs, illustrating factor effects. Orange indicates positive effects and blue indicates negative effects. The red “error line” represents the smallest 50% of the effects. Factors farthest from the red line have the greatest influence on the response and are retained according to effect size. As shown, the factors significantly influencing retention time are B–Gas Flow Rate, A–Column Temperature, and the AB interaction; factors affecting front peak resolution include B–Gas Flow Rate and C–Flow Ratio; factors affecting back peak resolution involve D–Multiple of Dilution; the factor influencing symmetry is A–Column Temperature; factors affecting the theoretical plate number are B–Gas Flow Rate, C–Flow Ratio, A–Column Temperature, and the BC interaction; factors affecting peak area are D–Multiple of Dilution, C–Flow Ratio, and CD. (D) The Box–Cox Plot for Power Transforms, based on the optimal lambda; this plot determines the appropriate power-law transformation. As shown, in this experiment all confidence intervals for lambda include 1, indicating that no additional transformation is required. (E) The Normal Probability Plot of Residuals; this plot assesses whether the residuals follow a normal distribution and helps evaluate the necessity of data transformation as well as potential outliers. As shown in the figure, this experiment did not exhibit any significant outliers. (F) The Residuals versus Predicted plot; this plot examines the assumption of constant variance and indicates whether residuals exhibit random scatter. It is used to detect large, biased deviations (beyond the red line). As shown, except for one data point in the R5 theoretical plate count, all residuals lie within the limits. Although it is common practice to ignore data points crossing the red line, it is retained here to respect the experimental data, and the residuals for this set are very close to the red line. (G) Cook’s Distance measures the influence of individual observations on the regression model. The x-axis represents the run order, which can reveal systematic changes (e.g., learning effects or fatigue). Encouragingly, Cook’s Distance values are randomly distributed with respect to Run Number and do not exceed the red line.

Based on comprehensive analysis, the Analytical Design Space and Method Operable Design Region were delineated: (H) Analytical Design Space. This plot shows how the key factors A–Column Temperature and B–Gas Flow Rate influence the six responses. Contour lines with curvature indicate that interactions also significantly affect the responses. (I) The Method Operable Design Region (MODR) delineates the constraint-compliant, feasible operational conditions for gas chromatography. The non-operational space is shaded in gray, the one-sided 95% confidence interval is shown in buff, and the feasible operational space is highlighted in bright yellow. The axes are oriented to the two factors most influential on the targeted response, with spatial constraints annotated accordingly.

**Table 8:** Confirmation location.

	Analysis	Predicted mean	Predicted median	Std dev	*n*	SE pred	95% PI low	Data mean	95% PI high
Point 1	Retention time	8.88825	8.88825	0.0199055	6	0.0128489	8.86025	8.878	8.91625
	Front resolution	1.78225	1.78225	0.334127	6	0.198845	1.35267	1.67783	2.21183
	Back resolution	6.56757	6.56757	2.8493	6	1.5852	3.14295	11.948	9.9922
	Symmetry factor	1.0375	1.0375	0.00479583	6	0.00259005	1.03194	1.039	1.04306
	Theoretical plates	75578.3	75578.3	2481.02	6	1717.41	71798.3	73633.5	79358.3
Point 2	Retention time	11.6875	11.6875	0.0199055	6	0.0128489	11.6595	11.6893	11.7155
	Front resolution	1.78225	1.78225	0.334127	6	0.198845	1.35267	1.71517	2.21183
	Back resolution	6.56757	6.56757	2.8493	6	1.5852	3.14295	——	9.9922
	Symmetry factor	1.0295	1.0295	0.00479583	6	0.00259005	1.02394	1.02617	1.03506
	Theoretical plates	85581.9	85581.9	2481.02	6	1717.41	81801.9	87539.1	89361.9

The analysis of the Method Operable Design Region involved randomly selecting two points for Confirmation Location validation (*n* = 6), as detailed in Table 8. For Point 1, all analyses, except for Back Resolution, which was unusually high, fell within the 95% confidence interval. However, since a higher Back Resolution is what we expect, we have chosen to overlook this anomaly. In Point 2, the target peak corresponds to the last peak in the chromatogram, resulting in the absence of recorded Back Resolution. All other analyses at Point 2 remained within the 95% confidence interval. Given that Point 2 shows superior resolution, it presents a lower risk of impacting quantification. Therefore, we have selected Point 2 as the fixed point for the analytical method and will proceed with evaluating the performance characteristics of the analytical procedure.

### Evaluation of analytical procedure performance characteristic

**Fig. 3:**
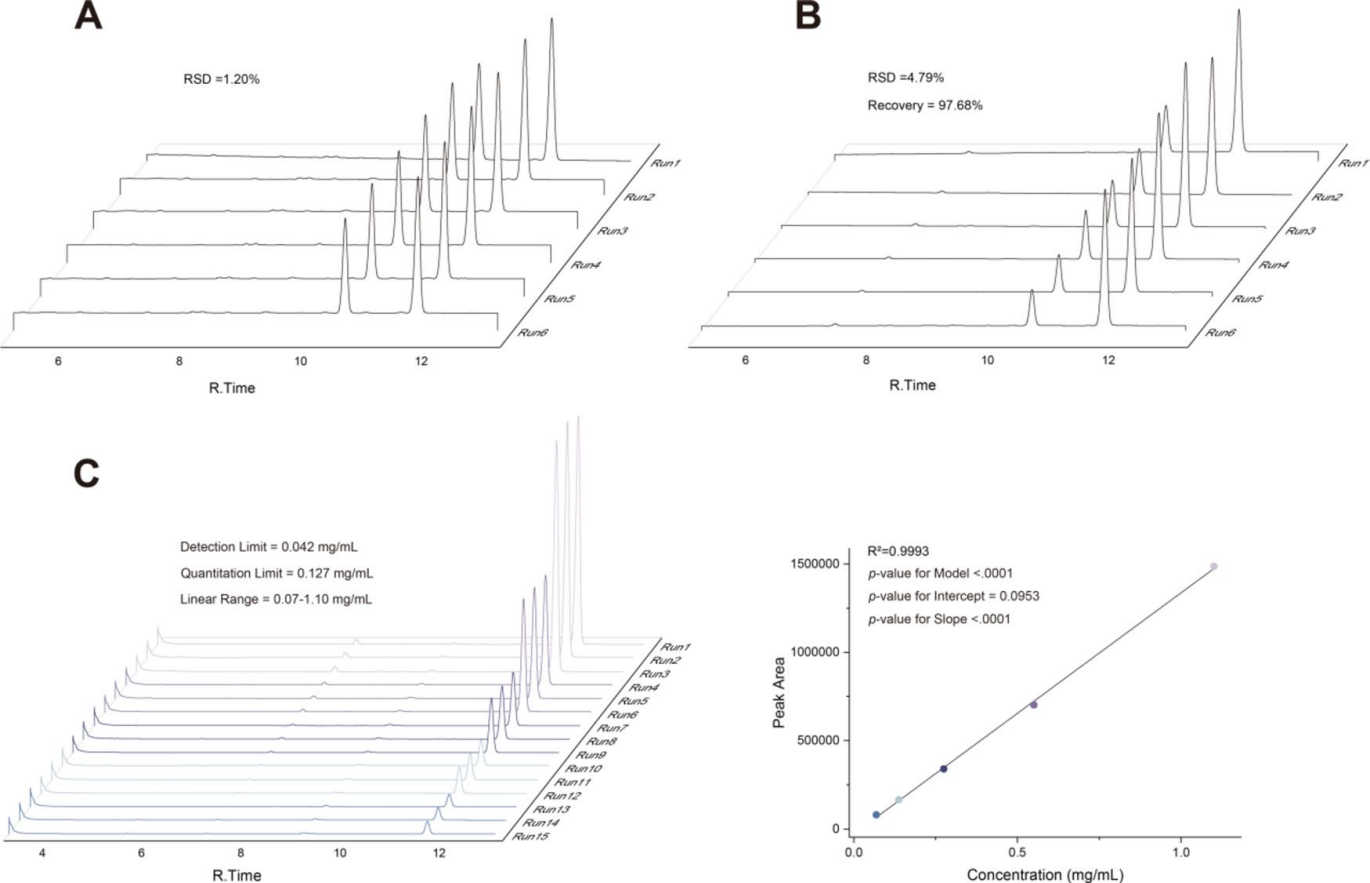
Evaluation of analytical procedure performance characteristic. Displays the evaluation of the analytical procedure’s performance characteristics. (**A**) Precision: Excellent repeatability was demonstrated with a relative standard deviation (RSD) of 1.20% (*n* = 6). (**B**) Accuracy: Spike recovery tests (*n* = 6) yielded a mean recovery of 97.68% with an RSD of 4.79%. (**C**) Linearity: A five-point calibration (each concentration in triplicate) produced a linear regression with R² = 0.9993. Statistical analysis indicated an insignificant intercept (*p* = 0.0953) and a highly significant slope (*p* < 0.001), both of which meet the acceptance criteria for pharmaceutical analysis. The maximum values of the z-axis (peak area) in the three plots (A, B, and C) are identical.

Following the optimization of the gas chromatography extraction parameters, the method’s performance was thoroughly evaluated. As shown in Fig. 3, The intra-day precision demonstrated acceptable variability, with a relative standard deviation (RSD) of 1.20% at 0.7 mg/mL, indicating good repeatability. The method exhibited excellent linearity over a range of 0.07 to 1.10 mg/mL (*R²* > 0.9993), with a statistically significant regression model (*p* < 0.0001) and an insignificant intercept (*p* = 0.0953). The limits of detection (LOD) and quantification (LOQ) for borneol were established at 0.042 mg/mL and 0.127 mg/mL, respectively. The accuracy was confirmed by a recovery of 97.68%, with an RSD of 4.79%. Collectively, these results demonstrate that the method is robust, precise, and reliable for accurate quantification of borneol.

### Accelerated destructive degradation tests, ADDT

**Table 9:** Model fitting.

Model	Response transformation	Distribution	Path definition $\:\mathcal{D}\left({\tau\:}_{i},{\chi\:}_{j},\beta\:\right)$	Log-likelihood	Number of parameters	Actual observations	AICc	BIC
Arrhenius rate	Log	Normal	$\:{\beta\:}_{0}-\mathrm{exp}\left({\beta\:}_{1}+{\beta\:}_{2}\cdot\:{\chi\:}_{j}\right)\cdot\:{\tau\:}_{i}$	84.46141	4	36	178.21	183.26
First-order kinetics type 3	Log	Normal	$\:{\beta\:}_{0}+\:{\beta\:}_{1}\cdot\:\mathrm{exp}(-{\beta\:}_{2}\cdot\:\mathrm{exp}({\beta\:}_{3}\cdot\:$ $(\frac{{E}_{\alpha\:}}{R}\cdot \:\frac{1}{25+273.15}-{\chi\:}_{j}))\cdot\:{\tau\:}_{i})$	84.468317	5	36	180.94	186.85
First-order kinetics type 4	Log	Normal	$\:{\beta\:}_{0}\cdot\:\mathrm{exp}(-{\beta\:}_{1}\cdot\:$ $\mathrm{exp}({\beta\:}_{2}\cdot\:(\frac{{E}_{\alpha\:}}{R}\cdot \:\frac{1}{25+273.15}-{\chi\:}_{j}))\cdot\:{\tau\:}_{i})$	92.508994	4	36	194.31	199.35

The fitting results for the inclusion complex data are presented in Table 9. Based on the log-likelihood of the negative log-likelihood, the corrected Akaike Information Criterion (AICc) and the Bayesian Information Criterion (BIC) were evaluated, indicating that the Arrhenius Rate model provides the best fit.

Table 10 presents the parameter estimates for both the encapsulated group and the physical mixture group, along with their standard errors. Additionally, it includes the 95% parameter confidence intervals based on the Wald method. The parameter estimates $\:{\beta\:}_{0}$, $\:{\beta\:}_{1}$ and $\:{\beta\:}_{2}$ describe the temperature-time relationship, while $\:{\upsigma\:}$ represents the scale parameter of the distribution. These estimates, derived from the collected ADDT data fitting, are used to simulate and evaluate a thermal index (TI) estimated through a statistical model^25^. This model involves an exponential function of the Arrhenius transformation multiplied by transformed time. setting the measurement scale for temperature at 25 °C.

**Table 10:** Parameter estimates.

Parameter	The inclusion complex				The physical mixture			
	Estimate	Standard error	Lower limit	Upper limit	Estimate	Standard error	Lower limit	Upper limit
$\:{\beta\:}_{0}$	4.6122	0.0054	4.6016	4.6229	4.4172	0.2281	3.9700	4.8643
$\:{\beta\:}_{1}$	245.41	88.82	71.328	419.4978	10.783	13.576	-15.8261	37.3917
$\:{\beta\:}_{2}$	-7.5952	2.703	-12.8930	-2.2975	-0.3735	0.416	-1.1889	0.4419
$\:{\upsigma\:}$	0.0255	0.003	0.0196	0.0314	0.6409	0.0755	0.4928	0.7889

**Fig. 4:**
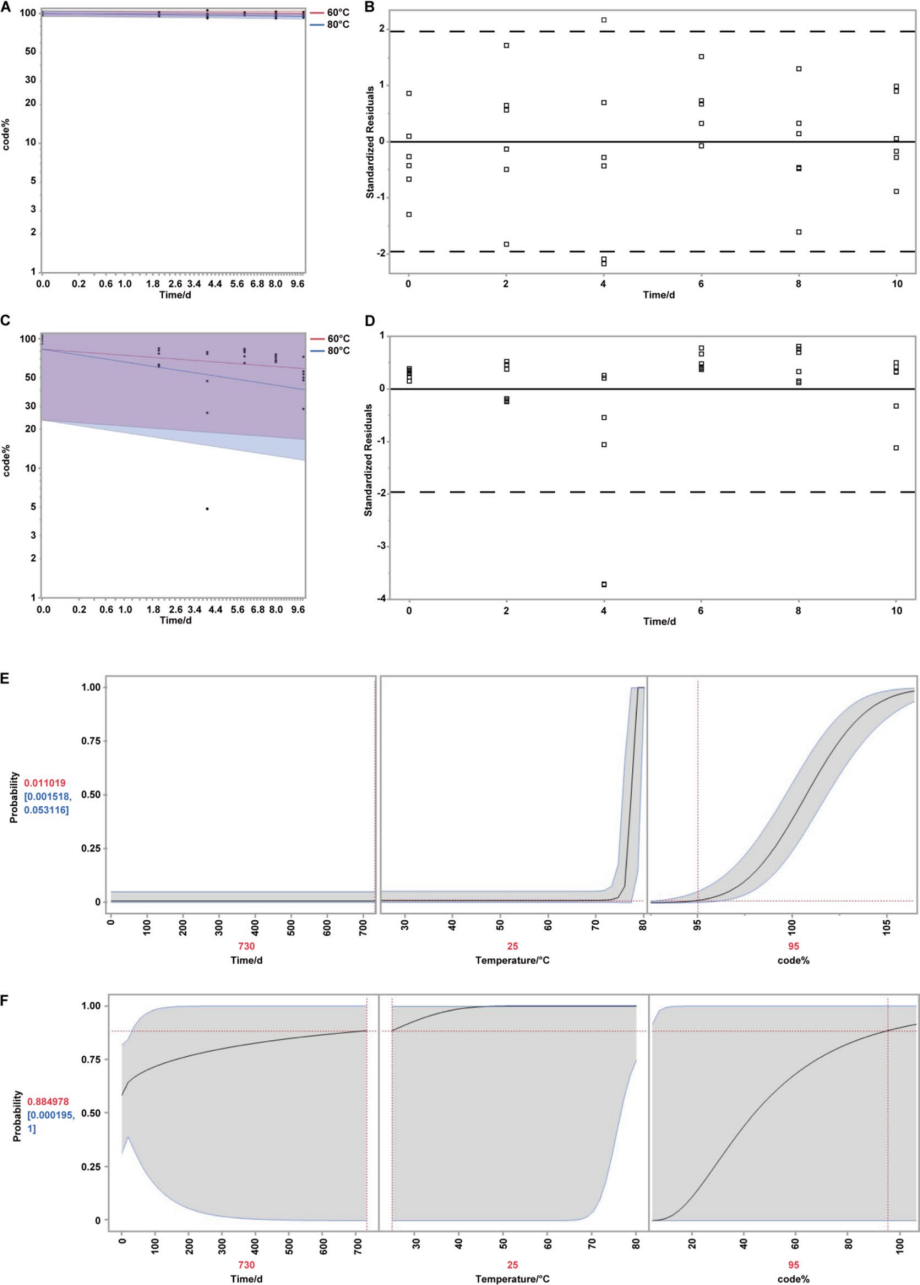
Accelerated destructive degradation tests. (**A**) Degradation trend of β-CD inclusion complex under accelerated factors (60 °C and 80 °C). (**B**) Distribution of residuals $\:{\epsilon\:}_{ijk}\:$during the experimental process. (**C**) Physical mixing used as a reference for stability assessment. (**D**) Distribution of residuals $\:{\epsilon\:}_{ijk}$​for physical mixing. (**E**) Crossing time distribution profiler for β-CD inclusion complex. (**F**) Crossing time distribution profiler for physical mixing.

As illustrated in Fig. 4, under the specified storage conditions of 25 °C and a shelf life of 730 days (2 years), with a residual content threshold of 95% (indicating a 5% loss as the criterion for failure), failure probabilities were characterized by using Crossing Time Distribution Profiler. The inclusion complex exhibited only a 1.10% probability of failure (defined as ≥ 5% content loss), in contrast to an 88.50% probability observed for the physical mixture. It is noteworthy that the synergistic protective effect of heat-sealed three-layer PET/AL/PE composite film bags should not be overlooked.

Natural essential oils are rich in low–molecular-weight hydrophobic compounds derived from primary and secondary plant metabolism, including terpenoids, aromatics, and hydrocarbons^30,31^. Due to their molecular characteristics, these components are highly volatile and prone to phase transitions with changes in temperature, pressure, and intermolecular interactions, leading to diffusion into the surrounding air and loss of the contents. Borneol, or related cyclic monoterpenes are readily sublimed and may exist in the gas phase at ambient temperature, which accounts for the observed decline in measurable concentration.

The encapsulation system constructs a highly closed microenvironment that minimizes the exposed surface area of entrapped molecules and restricts their contact with external factors such as oxygen, heat, and light^30^. β-Cyclodextrin interacts with specific essential oil molecules through external binding sites, hydrophobic cavities, and hydrogen bonding, reducing the accessibility of the encapsulated components and thereby enhancing their thermal stability and antioxidative properties^29,32^. Because temperature strongly influences the phase-transition–driven diffusion of volatile compounds, temperature was chosen as the accelerating factor in this accelerated destructive degradation study.

## Conclusion

In chromatographic analysis, the interactions among various factors are often complex, potentially involving higher-order interactions. Therefore, optimizing analytical methods through experimental design is essential. Interestingly, the results of this study reveal that the factors affecting the same response properties—specifically, the resolution of the target peak before and after separation—do not demonstrate similarities. The factors that significantly impact resolution prior to separation include the gas flow rate (*p* < 0.0001) and flow ratio (*p* = 0.0278); however, resolution after separation is predominantly influenced by the multiple of dilution (*p* = 0.0066). By employing experimental design, we can uncover the intricate patterns and combinations revealed by the objective results, allowing us to analyze significant factors at the data level and understand how these factors influence experimental outcomes. Based on fundamental chromatographic knowledge, we rationally analyze this outcome, specifically examining how the D-Multiple of Dilution affects Back Resolution. In the group with a multiple of dilution of + 1, the area of the undesired peak after the sample is below the minimum integration threshold, resulting in no automatic integration and a significant improvement in Back Resolution. In contrast, in the group with a multiple of dilution of -1, Back Resolution generally remains above 2.0, indicating no risk of affecting Back Resolution, and therefore, no further redundant studies are warranted.

The development of analytical methods is fundamentally a comparative process aimed at identifying how to achieve better results that meet expectations. Through experimental design and statistical analysis, we can scientifically streamline the comparative workload. Two-level factorial design, as a complete experimental approach, effectively illustrates how multiple factors influence the response and enables the analysis of higher-order interactions. However, when designing experiments with several influencing factors, a complete two-level factorial design is often impractical due to its exponential growth in the number of experimental runs, represented by 2^n^, where $\:n$ is the number of factors. In multi-factor experiments (more than three factors), a folded design is typically employed to reduce the number of trials. The trade-off with this approach is that some factors and their interactions may become aliased, making it difficult to discern their specific impacts on the response. Another limitation of two-level factorial designs, compared to response surface methodologies, lies in their exclusive focus on two-dimensional comparisons, lacking curvature analysis. In most scenarios, the influence of single factors on the response is linear, which may also result in saturation effects, where the influence varies, either diminishing or increasing, as the levels of the factors change. To address this, adding center points to the factor levels can improve fitting, though this increases the number of experimental runs from 2^n^ to 3^n^.

Robust experimental design requires well-defined objectives, efficient resource allocation, and rigorous parameter characterization. In industrial R&D with constrained resources, limiting experimental expenditure to ≤ 25% per study proves effective^33^. The strategic application of experimental design principles is crucial in this context. For research systems involving no more than three factors, two-level factorial designs demonstrate superior efficiency, provided that the factor levels are carefully selected. These levels must ensure the generation of observable effects while remaining scientifically and practically reasonable. The determination of such ranges should be grounded in preliminary experimental data, ensuring that effects significantly exceed measurement error and align with the realistic conditions of the system. For systems exhibiting nonlinear responses, adding center points can effectively upgrade a two-level factorial design to a response surface design, thereby facilitating more accurate modeling of curvature. However, such designs typically incur higher experimental costs. Throughout the study, rigorous residual analysis must be conducted to systematically identify outliers.

Through the rigorous development of analytical methods in accordance with the Q14 guidelines, we have achieved accurate and precise quantification of the samples. Notably, despite the presence of significant residuals (*p* > 2) in the mixed sample dataset—without any data censoring—we can confidently assert that the measured concentrations accurately reflect the sample contents. As a result, it is evident that the homogeneity of the physical mixture samples is inferior to that of the inclusion complex samples.

## Data Availability

Data sharing is not applicable to this paper. The data are provided only at the coded level or have been modified through a scale change. The actual levels must be kept confidential to protect proprietary information. Further inquiries can be directed to the corresponding authors.
